# Osteoarthritis of the temporomandibular joint in the Eastern Atlantic harbour seal (*Phoca vitulina vitulina*) from the German North Sea: a study of the lesions seen in dry bone

**DOI:** 10.1186/s12917-018-1473-5

**Published:** 2018-05-02

**Authors:** Catharina Ludolphy, Patricia Kahle, Horst Kierdorf, Uwe Kierdorf

**Affiliations:** Department of Biology, University of Hildesheim, Universitätsplatz 1, 31141 Hildesheim, Germany

**Keywords:** Temporomandibular joint, Osteoarthritis, Harbour seal, Lesion scoring, Wildlife disease

## Abstract

**Background:**

Pathological changes and resulting functional impairment of the temporomandibular joint (TMJ) can substantially affect physical condition, morbidity, and mortality of wildlife species. Analysis of TMJ disorders is therefore of interest for the characterization of the health status of populations of wild mammals. This paper, for the first time, analyses the prevalence of TMJ osteoarthritis (TMJ-OA) and the spectrum of osteoarthritic bone lesions of the TMJ in the Eastern Atlantic harbour seal (*Phoca vitulina vitulina*), applying a standardized scoring system. Dry skulls of 1872 individuals from the German North Sea, collected between 1961 and 1994, were examined for lesions consistent with a diagnosis of TMJ-OA. Of the skulls, 913 (48.8%) were from male, 959 (51.2%) from female seals, with age at death ranging from 2 weeks to 25 years. Possible associations of TMJ-OA with dental or periodontal disorders were also analysed.

**Results:**

Lesions consistent with TMJ-OA were found in 963 (53.9%) of the 1787 juvenile/subadult (5 weeks to 5 years of age) and adult (> 5 years) specimens, the condition mostly (95.0% of affected individuals) occurring in a bilateral fashion. Males were affected more frequently than females (*p* < 0.001), while lesion severity tended to be higher in females (*p* < 0.05). Severity of TMJ-OA lesions was positively correlated with age (*p* < 0.001). Lesion severity was also weakly positively correlated with the number of fractured teeth (*p* < 0.05) and of intravitally lost teeth (*p* < 0.01), when controlling for age at death as a confounder.

**Conclusions:**

TMJ-OA is a common disorder in the Eastern Atlantic harbour seal. The more pronounced severity of the lesions in females compared to males is basically attributed to the higher average age of the female subsample. The causes underlying the high prevalence of TMJ-OA in the studied assemblage remain unknown. Most of the specimens (75.3%) analysed in the present study were found dead during the first phocine distemper virus epizootic in 1988. Therefore, it is assumed that, contrary to other museum collections, only little overrepresentation of pathological skeletal conditions is present in this death sample compared with the population from which it originated.

**Electronic supplementary material:**

The online version of this article (10.1186/s12917-018-1473-5) contains supplementary material, which is available to authorized users.

## Background

The temporomandibular joint (TMJ) is a defining feature of the class Mammalia. It is a synovial articulation between the head (*caput mandibulae*) of the mandible’s condylar process and the mandibular fossa (*fossa mandibularis*) of the squamous part of the temporal bone [1–4]. The cavity of the TMJ is separated into a dorsal and a ventral compartment by an articular disc that is an inward extension of the fibrous joint capsule [1, 4–6]. While the articular surfaces of most synovial joints are covered with hyaline cartilage, those of the TMJ are lined by fibrous tissue [4, 7, 8]. Underneath this fibrous layer is a cell-rich (proliferative zone) that itself is underlain by fibrocartilage [4, 7, 8]. The distinctness and the thickness of the cell-rich and the fbrocartilaginous layers vary between different articular areas and also with age [4, 7, 8]. It has, for instance, been shown in different species, including the California sea lion (*Zalophus californianus*), that the mandibular fossa has a thinner layer of fibrocartilage than the mandibular head [4, 7, 8]. The reason for this may be differences in the loading pattern of the two components [8]. In the mandibular head, the deeper zone of the fibrocartilage undergoes mineralization, and the subchondral bone is therefore overlain by a zone of calcified (mineralized) cartilage, which is more highly mineralized than the adjacent bone [4]. Anatomy and functional mechanics of the TMJ vary among species, depending on the different requirements for mastication [1, 4, 9]. In carnivorans, such as seals, the movement of the TMJ is primarily hinge-like [5].

Recently there has been increasing interest in the pathology of the TMJ of different pinniped species [8–13] and other carnivorans [14–17]. The findings of these studies are of relevance not only from a comparative point of view but also for management and conservation, as pathological alterations and resulting functional impairment of the TMJ are considered important factors affecting physical condition, morbidity, and mortality of wildlife species [11, 13]. Analysing prevalence and severity of TMJ disorders can therefore substantially contribute to the characterization of the overall health status of mammal populations.

Studies in different species revealed osteoarthritis (OA) as the most common disease affecting the TMJ [12, 18]. Osteoarthritis, also referred to as osteoarthrosis or degenerative joint disease, is a primarily degenerative disease that can, however, be accompanied by secondary inflammatory changes [12, 18, 19]. The pathophysiology of OA is complex, with a variety of interacting risk factors [19–21]. Sex, systemic illness, traumatic and other external impacts can influence occurrence and individual expression of the disease [19, 22]. The strongest predictor for the development of OA is, however, individual age [23]. Disease progression in TMJ-OA is characterized by increasing deterioration of articular cartilage and the articular disc as well as pathological changes of the subchondral bone at more advanced stages [18]. Another common feature in the case of OA is the formation of periarticular osteophytes [12, 19, 24]. The progression of OA is thought to be accelerated by an increased force transmission or modified load patterns [25]. Pain, instability, and loss of function of the TMJ can lead to a considerable impairment of food intake and a resulting weakening of affected individuals, and can thereby cause increased morbidity and mortality [12, 18, 21].

Studies on different pinniped species revealed marked interspecific variation in the prevalence and severity of TMJ-OA [10–13]. These studies were performed on dry skulls (museum specimens) of animals collected in various regions of North America. Thus far, systematic surveys on TMJ pathology in pinnipeds from Europe have not been conducted.

The harbour seal (*Phoca vitulina*) has the broadest geographical distribution of all seal species, inhabiting coastal areas of the Holarctic region [26, 27]. There are currently five recognized subspecies of the harbour seal [27]. In Europe, the Eastern Atlantic harbour seal (*Phoca vitulina vitulina*) occurs from the northern part of the Iberian Peninsula, along the coasts of mainland Europe, the United Kingdom, Ireland, and Iceland, to the Barents Sea in north-western Russia and north to Svalbard [28]. The population of the Eastern Atlantic harbour seal has more recently been estimated at approximately 113,450 to 134,200 individuals [29]. In 2016, approx. 26,000 individuals were counted in the Wadden Sea that is part of the North Sea [30]. In 1988 and in 2002, two phocine distemper virus (PDV)-epizootics severely decimated the population of the Eastern Atlantic harbour seal [31]. However, the population recovered quickly and its overall health improved since the mid-1970s [32]. A study of 355 carcasses of harbour seals collected between 1996 and 2005 along the coast of Schleswig-Holstein, Germany, which were either found dead or killed due to severe illness, found bronchopneumonia to be the most common cause of death [33]. Other findings in the seals were traumatic lesions, gastroenteritis, uterine torsion or dystocia, polyarthritis/polymyositis, intestinal torsion, septicaemia, dermatitis, and keratitis. Human activities negatively affecting the population of the Eastern Atlantic harbour seal include pollution, fisheries, shipping traffic, tourism, gravel extraction, and the building and operation of offshore windmill farms [32].

Harbour seals are small to medium-sized phocids with no obvious sexual dimorphism [34, 35]. Adult males are only slightly larger (average length 160 cm) and heavier (average body mass 75 kg) than adult females (150 cm, 67 kg) [36]. Female harbour seals attain sexual maturity at 3 to 4, males at 4 to 6 years of age [36]. Females are fully grown at 6 to 7, males at 7 to 9 years [27]. Maximum lifespan of harbour seals is 35 years, with females normally achieving a higher age than males [29].

The harbour seal is an opportunistic predator that feeds on a variety of small to medium-sized fish, squid, and crustacean species [27, 35]. The harbour seal’s dentition is adapted to catch and hold its prey, which is swallowed largely unchewed [35]. Accordingly, the muscles of mastication are rather weak [37]. The permanent dentition (dental formula: I 3/2, C 1/1, P 4/4, M 1/1) of the harbour seal comprises 34 teeth and is fully functional after the relatively short suckling period of 4 to 6 weeks [38–40]. The deciduous teeth are mostly already lost in utero [38, 39].

The present study analysed the prevalence and severity of lesions consistent with a diagnosis of TMJ-OA in a large sample of dry skulls of the Eastern Atlantic harbour seal from the collection of the Zoological Institute and Museum of the University of Kiel, Germany (ZIK). In a previous investigation [41], age at death had been determined in all studied individuals based on morphological criteria (individuals ≤1 yr) or analysis of cementum annulation in canine teeth (individuals > 1 yr). A pilot study on a small subsample of this material had reported the occurrence of various lesions in the bony components of the TMJ [42], but no systematic investigation had been undertaken. The results of the present study are compared with findings previously obtained in another subspecies, the Eastern Pacific harbour seal (*Phoca vitulina richardii*) [11]. In addition, possible associations between TMJ-OA and dental or periodontal pathologies are analysed.

## Results

**Fig. 1:**
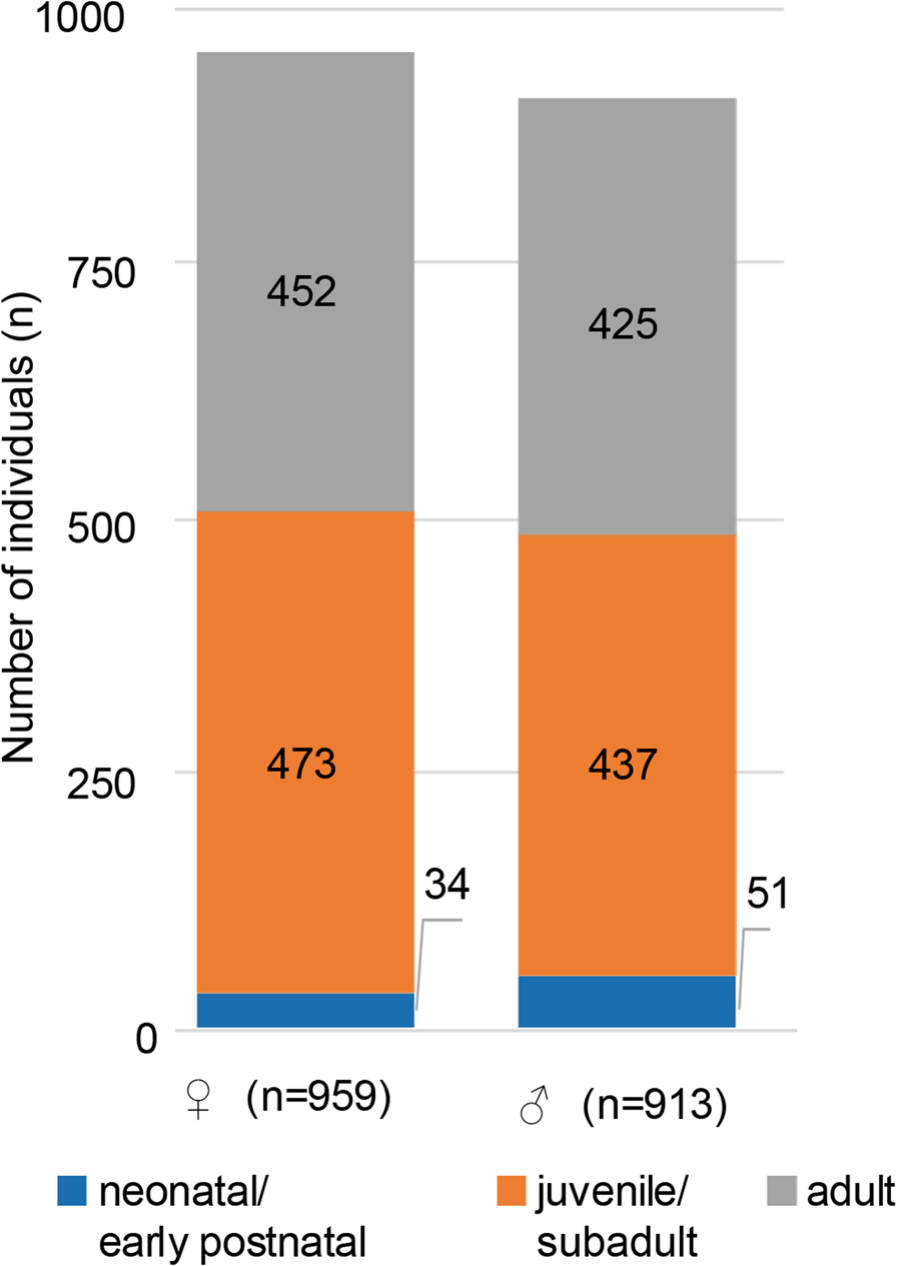
Numbers of examined individuals of *Phoca v. vitulina* in the different age classes; left: females, right: males

**Fig. 2:**
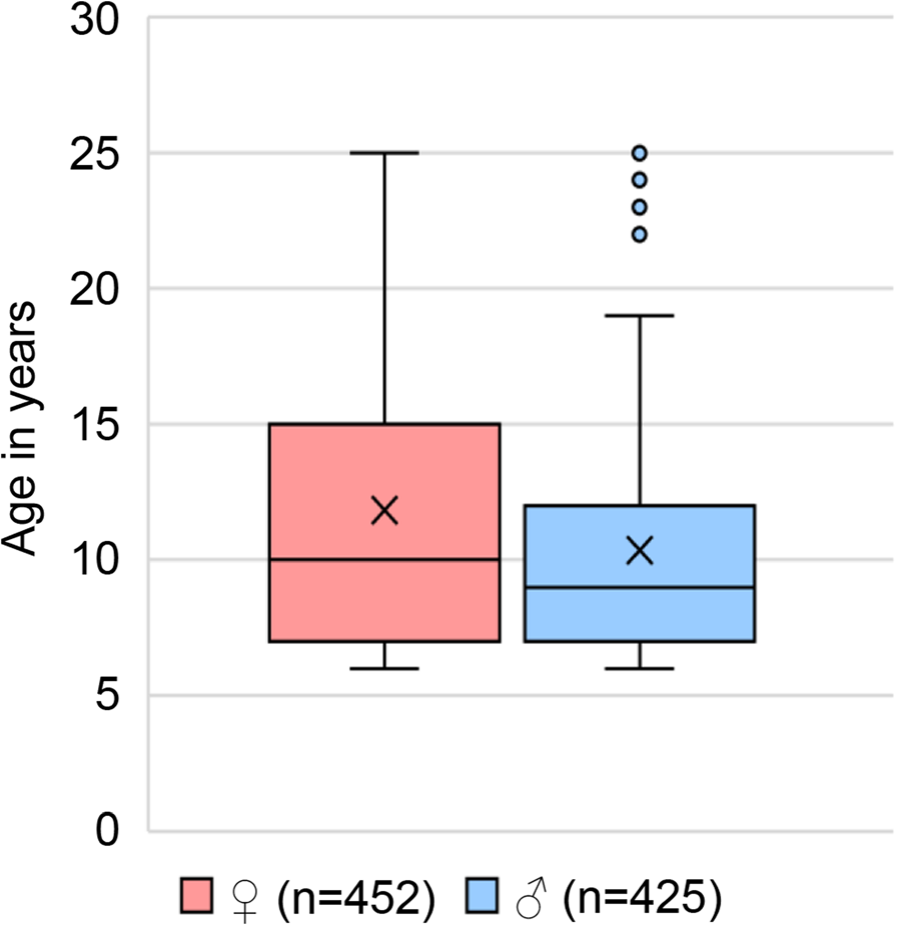
Distribution of age at death of adult female and male individuals (*n* = 877). Box-Whisker-Plots show median (line in box), mean (x), interquartile range (box), non-outlier range (1.5-fold interquartile range, whiskers) as well as outliers (points)

Of the 1872 skulls examined in this study, 913 (48.8%) were from males and 959 (51.2%) from females (Fig. 1). Age-at-death comparison between males and females (juvenile/subadults and adults pooled) revealed significant differences between sexes (*t*_(1751.738)_ = − 2.667, *p* = 0.008). While there existed no significant sex difference in age between juvenile/subadult females and males (*t*_(908)_ = − 0.735, *p* = 0.463), a significant sex difference in age was found for adult animals (*t*_(822.676)_ = − 4.409, *p* < 0.001), with higher mean, median, and maximum ages in females (Fig. 2).

None of the 85 skulls of neonatal/early postnatal (≤ 4 weeks) individuals was diagnosed with TMJ-OA. They were therefore excluded in the group comparisons, reducing the overall number of skull specimens to 1787 in these analyses. However, when calculating correlations with age at death, all 1872 studied skulls were included.

**Fig. 3:**
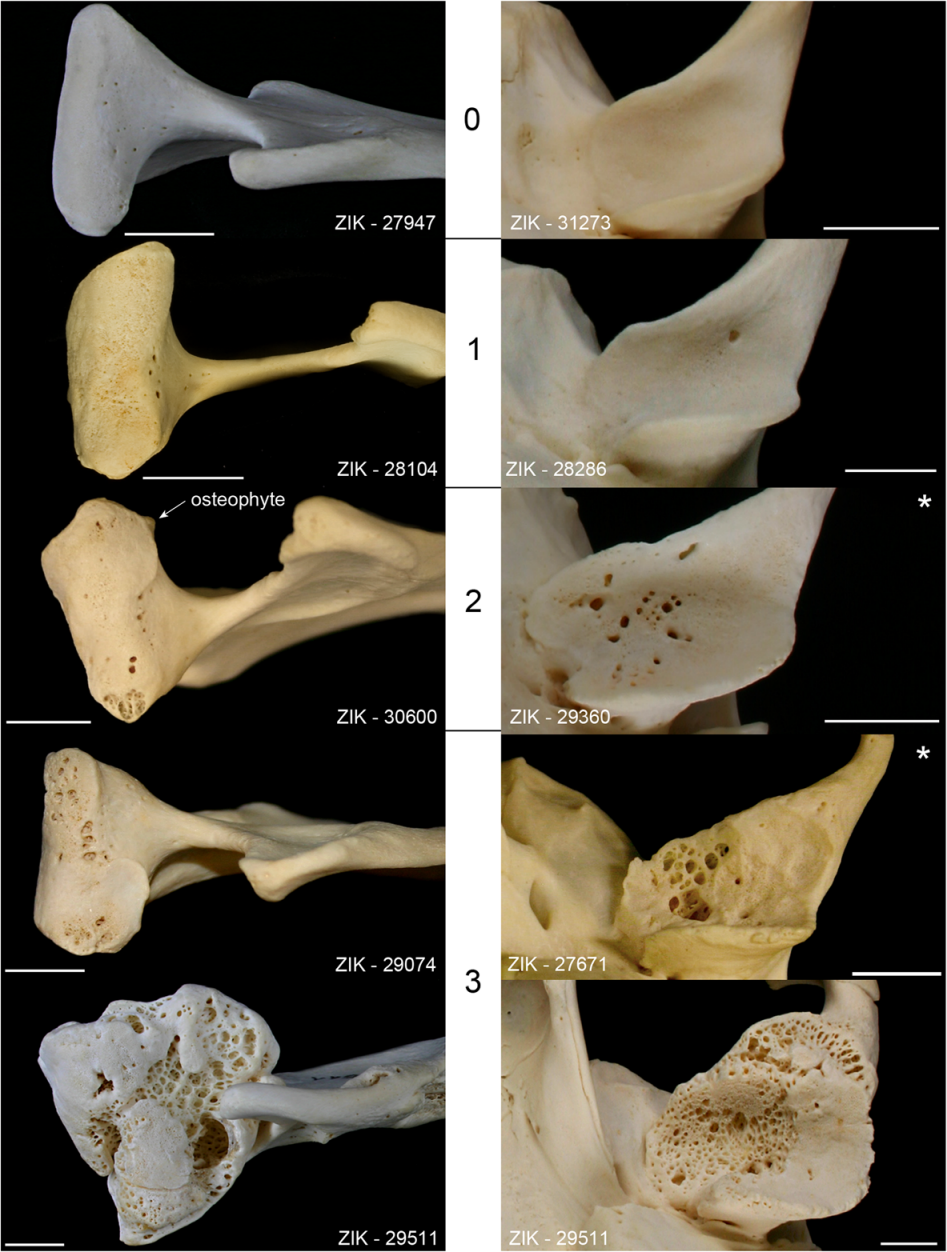
Illustration of gross TMJ-OA lesions in Eastern Atlantic harbour seals (*Phoca v. vitulina*) from the collection of the ZIK. The left row shows mandibular heads and the right row mandibular fossae, demonstrating lesion scores 0 to 3. Bars indicate 1 cm; * mirrored image to facilitate comparison

The normal condition of the mandibular head and the mandibular fossa and the spectrum of gross osteoarthritic lesions observed in the studied harbour seal skulls by external inspection are illustrated in Fig. 3. The youngest individual exhibiting lesions consistent with TMJ-OA was a two-month-old male. The oldest individuals free of such lesions were two 11-year-old females. Of the 1787 individuals from the age classes juvenile/subadult and adult, 963 (53.9%) exhibited lesions consistent with TMJ-OA on at least one articular surface. Of the total number of 7148 TMJ articular surfaces, 2754 (38.5%) were affected by OA. In 166 (17.2%) of the 963 skull specimens showing TMJ-OA lesions, only one articular surface was affected, the lesions being mostly (92.8%) mild (score 1). Two articular surfaces were affected in 256 skulls (26.6%), three in 88 skulls (9.1%) and all four articular surfaces in 453 skulls (47.0%). The most common articular surface affected was the left mandibular fossa (712 of 2754 articular surfaces showing osteoarthritic lesions, 25.9%), followed by the right mandibular fossa (697 cases, 25.3%).

**Fig. 4:**
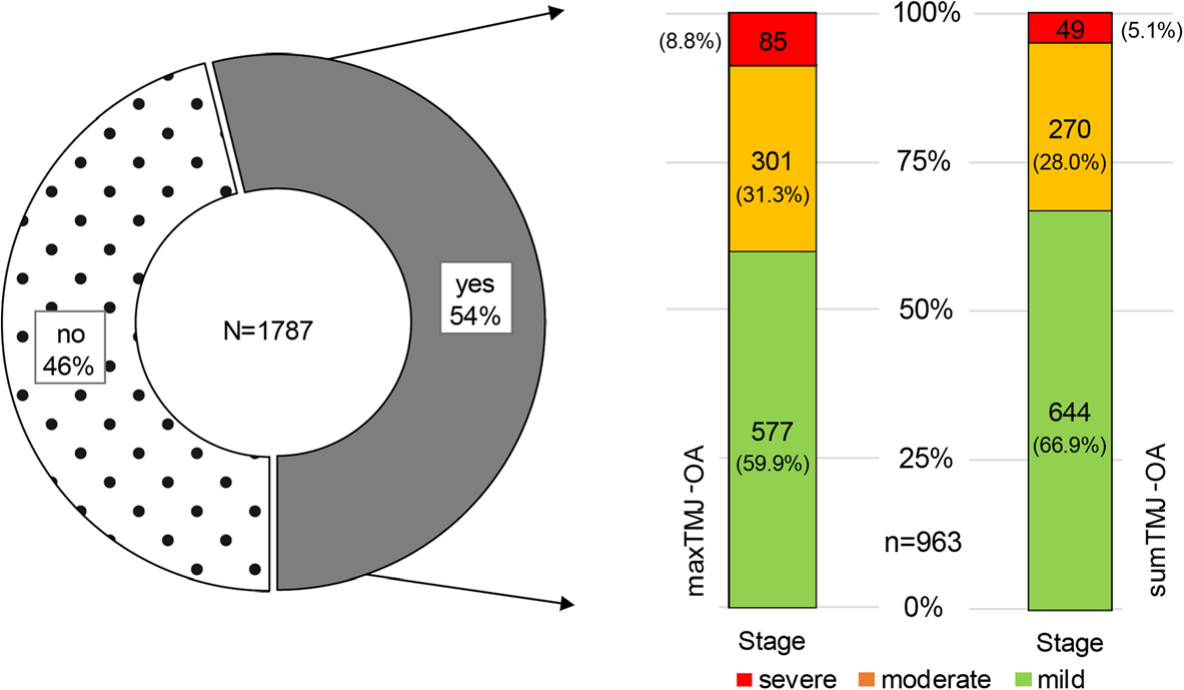
Frequency and severity of TMJ-OA lesions in the death-sample (juvenile/subadult and adult individuals). No = No lesions of TMJ-OA present, Yes = lesions of TMJ-OA present

Eighty-five (8.8%) of the 963 skulls affected by TMJ-OA exhibited severe osteoarthritic lesions (score 3) on at least one articular surface. Only 9 (0.9%) of the 963 affected skulls showed score-3-lesions on all four surfaces. Using sumTMJ-OA instead of maxTMJ-OA as the parameter for assessing severity of articular changes reduced the frequency of moderate and severe cases and increased that of mild TMJ-OA (Fig. 4).

Overall, 507 male individuals (58.8% of all juvenile/subadult and adult males) showed lesions of TMJ-OA, compared to 456 female individuals (49.3% of all juvenile/subadult and adult females) (Table 1).

**Table 1:** Frequency and severity of TMJ-OA lesions in male and female (juvenile/subadult and adult) individuals

		maxTMJ-OA				sumTMJ-OA			
	Stage	Males		Females		Males		Females	
Frequencies (*n* (%))	normal	355	(41.2%)	469	(50.7%)	355	(41.2%)	469	(50.7%)
	mild	326	(37.8%)	251	(27.1%)	364	(42.2%)	280	(30.3%)
	moderate	145	(16.8%)	156	(16.9%)	126	(14.6%)	146	(15.8%)
	severe	36	(4.2%)	49	(5.3%)	17	(2.0%)	30	(3.2%)
	total	862	(100%)	925	(100%)	862	(100%)	925	(100%)
Percentiles	25th	normal		normal		normal		normal	
	50th	mild		normal		mild		normal	
	75th	mild		mild		mild		mild	
	90th	moderate		moderate		moderate		moderate	
	95th	moderate		severe		moderate		moderate	
	99th	severe		severe		severe		severe	

The difference in the prevalence of TMJ-OA between sexes was statistically significant (χ^2^_(1)_ = 16.272, *p* < 0.001; odds ratio of 1.47 [95% CI: 1.22–1.77] for males:females). On average, females exhibited more severe lesions than males, the difference between sexes again being statistically significant (Table 2).

**Table 2:** Comparison of TMJ-OA lesion severity of affected individuals (at least one articular surface with a score > 0) between sexes in the different age classes as well as in the whole sample (Mann-Whitney U-Tests)

			*N*_*affected*_	*%*_*affected*_	*M*	*Mdn*	*mean rank*	*U*	*z*	*p*	*d*
Pooled	max TMJ-OA	male	507	58.8	1.43	1	459.43				
		female	456	49.3	1.56	1	507.10				
		total	963	53.9				104,152.0	−3.059	0.0022	0.172
	sum TMJ-OA	male	507	58.8	1.32	1	457.03				
		female	456	49.3	1.45	1	509.77				
		total	963	53.9				102,935.0	−3.567	0.0004	0.190
Juvenile/Subadult	max TMJ-OA	male	125	28.6	1.18	1	105.13				
		female	98	20.7	1.38	1	120.76				
		total	223	24.5				5266.5	−2.590	0.0096	0.242
	sum TMJ-OA	male	125	28.6	1.07	1	107.66				
		female	98	20.7	1.16	1	117.53				
		total	223	24.5				5583.0	−2.151	0.0315	0.152
Adult	max TMJ-OA	male	382	89.9	1.51	1	356.97				
		female	358	79.2	1.61	1	384.94				
		total	740	84.4				63,208.0	−1.997	0.0458	0.131
	sum TMJ-OA	male	382	89.9	1.40	1	351.51				
		female	358	79.2	1.53	1	390.77				
		total	740	84.4				61,122.5	−2.893	0.0038	0.184

TMJ-OA is more frequent in males. Conversely, higher mean score (M) and mean rank indicate that females are more severely affected. In all age groups (juveniles/subadults only, adults only, both groups pooled) the difference between sexes is significant (*p* < 0.05). However, the effect size (Cohen’s d) is always rather small. Mdn – median score

Of the 877 adult individuals, 84.4% exhibited lesions consistent with TMJ-OA, while this was the case in only 24.5% of the 910 juvenile/subadult individuals (Table 3). The difference in the frequency of TMJ-OA between juvenile/subadult and adult individuals was statistically significant (χ^2^_(1)_ = 644.281, *p* < 0.001; odds ratio of 16.64 [95% CI: 13.13–21.09] for adults:juveniles/subadults). There was also a significant difference in the severity of lesions between the two age classes with more severe lesions present in adults (maxTMJ-OA: *U* = 61,875.00, *z* = − 6.528, *p* < 0.001; sumTMJ-OA: *U* = 57,794.50, *z* = − 8.242, *p* < 0.001). Rank correlation revealed a strong positive relationship between age at death (years) and maxTMJ-OA (*r*_s_ = 0.671, *p* < 0.001) as well as sumTMJ-OA (*r*_s_ = 0.689, *p* < 0.001).

**Table 3:** Frequency and severity of TMJ-OA lesions in the age classes juvenile/subadult and adult

	maxTMJ-OA		sumTMJ-OA	
Stage	Juvenile/Subadult	Adult	Juvenile/Subadult	Adult
Normal	687 (75.5%)	137 (15.6%)	687 (75.5%)	137 (15.6%)
Mild	179 (19.7%)	398 (45.4%)	200 (22.0%)	444 (50.6%)
Moderate	28 (3.1%)	273 (31.1%)	21 (2.3%)	251 (28.6%)
Severe	16 (1.8%)	69 (7.9%)	2 (0.2%)	45 (5.1%)
Total	910 (100%)	877 (100%)	910 (100%)	877 (100%)

Like the severity of TMJ-OA, also the severity of periodontal disease increased with age at death of the harbour seals (Kahle et al., unpublished work). Severities of TMJ-OA and periodontal lesions were, however, uncorrelated when controlling for age at death as a confounder (Table 4). In contrast, rank correlation analysis revealed small but significant positive relationships between TMJ-OA lesion severity and the number of fractured teeth as well as the number of intravitally lost teeth (Table 4).

**Table 4:** Partial rank correlations, with age at death (yr) as confounder, of periodontal and dental pathologies with TMJ-OA lesion severity (all three age classes, *n* = 1872)

	Periodontitis^a)^	No. of tooth fractures	No. of intravitally lost teeth
maxTMJ - OA	0.029^ns^	0.048*	0.096**
sumTMJ - OA	0.005^ns^	0.052*	0.112**

^a)^maxTMJ-OA with maxPeriodontitis; sumTMJ-OA with sumPeriodontitis

** *p* ≤ 0.01* 0.01 > *p* < 0.05, ^ns^ *p* ≥ 0.05

**Fig. 5:**
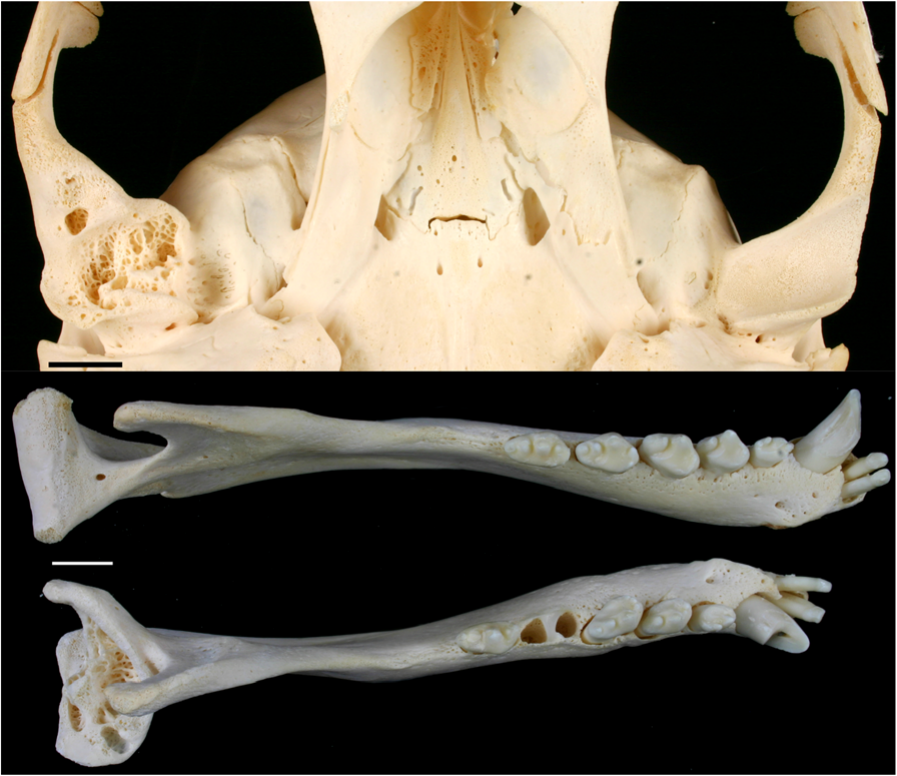
Mandibular fossae (top) and mandibular heads (bottom) of a four-year-old male harbour seal (ZIK - 29099) with asymmetric TMJ-OA. The right head and fossa exhibit severe (score 3) osteoarthritic lesions, whereas the left TMJ appears normal (score 0 for both, the mandibular head and the mandibular fossa). Bars indicate 1 cm

**Fig. 6:**
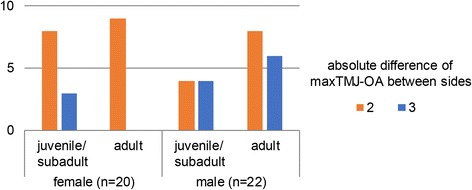
Absolute difference between sides (Δ dex/sin) in lesion score in the case of asymmetric TMJ-OA. The total number of individuals exhibiting asymmetric changes was 42

Asymmetric TMJ-OA (Fig. 5) was diagnosed in 42 out of 963 affected individuals (4.4%, 22 males, 20 females). Males tended to show more pronounced differences between sides than females (Fig. 6), whereas no obvious difference was apparent between the age classes juvenile/subadult and adult.

**Fig. 7:**
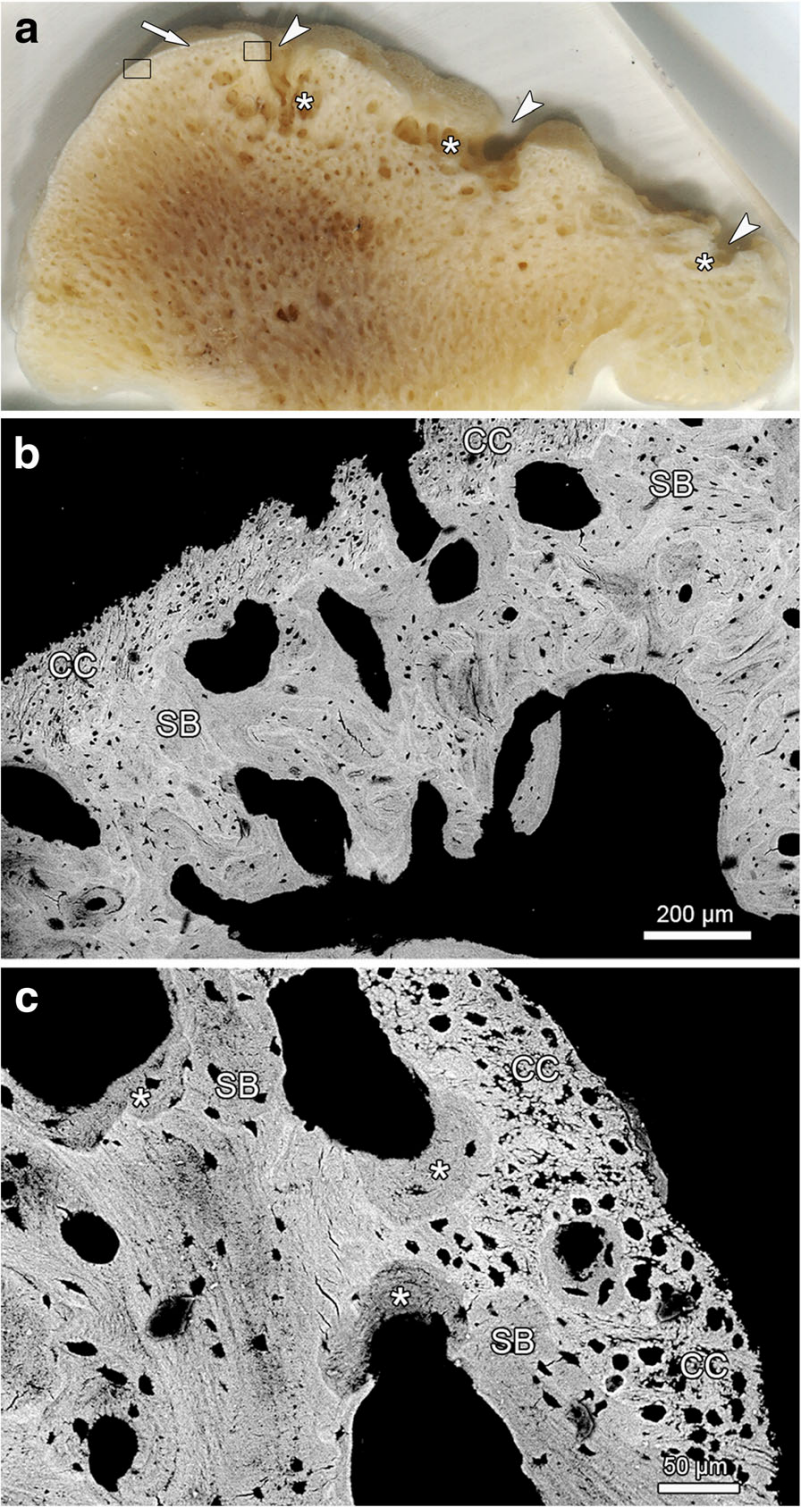
Sagittally sectioned mandibular head of left mandible of individual ZIK - 29511 showing severe (score 3) osteoarthritic changes. **a** gross appearance of sectioned mandibular head, anterior to the right. Note marked porosity of subchondral bone with presence of subchondral cysts of different size (asterisks), some of which open onto the articular surface (arrowheads); arrow: layer of calcified fibrocartilage; rectangles indicate the approximate location of the regions shown in (**b**) and (**c**). **b** BSE-image showing calcified fibrocartilage (CC) and subchondral bone (SB) of the mandibular head. Note marked porosity and mottling of subchondral bone. **c** BSE-image showing calcified fibrocartilage (CC) and subchondral bone (SB) at higher magnification. Note signs of increased remodeling of the SB and bone mottling. Asterisks: former resorption bays that have been filled in with new bone

Analysis of the sectioned mandibular head from an adult individual (ZIK - 29511) with severe osteoarthritic lesions (score 3) showed that the subchondral bone was still covered by a thin layer of calcified fibrocartilage (Fig. 7a-c), which had not been removed during the maceration process. On the BSE images, the mineralized matrix of the calcified fibrocartilage appeared slightly brighter than that of the subjacent bone, indicating the former’s higher degree of mineralization (Fig. 7b, c). The chondrocyte lacunae in the calcified fibrocartilage were more numerous than the osteocyte lacunae in the subchondral bone (Fig. 7b, c). The subchondral bone showed increased porosity and multiple cystic lesions of varying size. Some of these subchondral cysts opened onto the articular surface and had thus presumably communicated with the articular cavity (Fig. 7a). BSE imaging revealed signs of increased remodelling and related porosity of the subchondral bone (Fig. 7b, c). Due to the presence of more highly mineralized, older bone (appearing brighter in the BSE images) and less mineralized, younger bone (appearing darker), the subchondral bone showed a distinct mottling (Fig. 7b, c).

Table 5 compares frequency of TMJ-OA and lesion severity between the two subspecies of harbour seals (*P. v. vitulina* [this study] and *P. v. richardii* [11]). The Eastern Pacific harbour seal was less frequently but more severely affected by TMJ-OA (46% of the affected articular surfaces scoring moderate or severe) than the Eastern Atlantic harbour seal (32%) (Table 6). The differences in TMJ-OA prevalence (χ^2^_(1)_ = 26.260, *p* < 0.001) and lesion severity (χ^2^_(3)_ = 152.757, *p* < 0.001) between the two subspecies were statistically significant.

**Table 5:** Comparison of frequency (individual-based) and lesion severity (based on articular surfaces) of TMJ-OA between the two subspecies *Phoca v. richardii* ([10] and *Phoca v. vitulina* [this study]. Only juvenile/subadult and adult individuals

	*P. v. richardii*	*P. v. vitulina*
Frequency (individuals)		
TMJ-OA yes	67 (34.5%)	963 (53.9%)
TMJ-OA no	127 (65.5%)	824 (46.1%)
Total	194 (100%)	1787 (100%)
Stage (articular surfaces)		
Normal	642 (82.7%)	4394 (61.5%)
Mild	72 (9.3%)	1875 (26.2%)
Moderate	38 (4.9%)	715 (10.0%)
Severe	24 (3.1%)	164 (2.3%)
Total	776 (100%)	7148 (100%)

## Discussion

Recently, osteoarthritic changes of the TMJ were included in several studies on oral health of marine and terrestrial carnivorans from North America [10–17]. In contrast, comprehensive studies on European (sub-)species of Carnivora have, to our knowledge, not previously been performed. Thus far, only Stede & Stede [42] described lesions of the TMJ in a small sample of harbour seals skulls from the German North Sea in a qualitative way. The present study therefore, for the first time, obtained and analysed data on lesions consistent with TMJ-OA in a large sample of Eastern Atlantic harbour seal skulls in a standardised manner. Moreover, potential relationships of TMJ-OA with pathological changes in the dentition were evaluated for the first time. Finally, using previously published data on the Eastern Pacific harbour seal [11], the prevalence and severity of TMJ-OA were compared between that subspecies and the Eastern Atlantic harbour seal.

The gross lesions of TMJ-OA observed by external inspection in the harbour seals ranged from minor unevenness and pitting of the articular surface to severe destruction and disfigurement of the mandibular condyle and the mandibular fossa, with the spectrum of changes basically corresponding to that described in other pinniped species [10–13]. The changes seen in the sectioned mandibular head matched those observed macroscopically and histologically in cases of OA both in the TMJ and other synovial joints of mammals [19, 24, 43–45]. A layer of calcified fibrocartilage was still present in the studied mandibular head. This indicates that calcified fibrocartilage may be retained during maceration and can therefore still be present in dry skeletons. Corresponding findings were previously reported for tendon attachment sites of dry human bones from adult individuals [46].

Assessing the occurrence of TMJ-OA based on the analysis of dry skulls can lead to an underestimation of the prevalence of the condition in the source population, as early stages of OA that affect only the articular cartilage but not the subchondral bone [47] cannot be diagnosed. In contrast, the evaluation of death-stranded individuals may result in an over-representation of individuals with skeletal pathology (including lesions of TMJ-OA) in the death sample compared to the source population [48]. However, in the present study most of the individuals analysed had died during a single, virus-related mass mortality event, viz., the PDV-epizootic of 1988. The clinical manifestations of a PDV-infection and the frequently observed secondary infections with other pathogens suggest a rather rapid death of infected individuals [49]. It is, therefore, assumed that many harbour seals died independently of their skeletal health condition. This leads us to conclude that with respect to the prevalence and severity of TMJ-OA, the present death sample is probably more representative of the original population than would have been the case if the death sample had consisted of stranded individuals collected over a period of many years or even decades.

With 963 individuals (53.9%) of the 1787 juvenile/subadult and adult individuals and 2754 (38.5%) out of 7148 articular surfaces affected, TMJ-OA is a common condition in the Eastern Atlantic harbour seal. The evaluation of four articular surfaces per skull, which was performed in the present study, in principle allows for a regionally detailed analysis of TMJ-OA. However, when doing this it must be borne in mind that variation in the aetiology of TMJ-OA between affected joint surfaces of an individual may exist [22].

The present study found significant differences between sexes regarding prevalence of TMJ-OA as well as lesion severity, with males being more often and females slightly more severely affected. The more severe affection of the TMJ in females may be attributed to the higher average age of the female compared to the male subsample. The higher prevalence of TMJ-OA in males could be related to a higher loading of the TMJ in males compared to females, including a higher strain on the joint due to inter-male fighting. Higher proportions of fractured teeth and of intravitally lost teeth in male compared to female harbour seals (Kahle et al., unpublished work), and the effects of these dental abnormalities on the occlusal pattern and the jaw mechanics, may also contribute to the higher frequency of TMJ-OA in males. Similar results were obtained for the walrus (*Odobenus rosmarus*), where breeding males are known to use their teeth and jaws aggressively as weapons, potentially resulting in higher proportions of dental fractures and higher loads on the TMJ [13].

As expected, adult individuals were more often and more severely affected by TMJ-OA than juveniles/subadults. The positive relationship between age and lesion severity (both maxTMJ-OA and sumTMJ-OA) was strong, revealing age as an important predictor for the severity of osteoarthritic lesions of the TMJ in seals.

Previous studies on various pinniped species, also evaluating the condition in dry skulls, found partly lower and partly higher frequencies of TMJ-OA compared with the results in *P. v. vitulina* (Table 6). Compared to our findings in the Eastern Atlantic harbour seal, the severity of TMJ-OA observed in other pinnipeds tended to be higher, especially in the case of the Northern fur seal (*Callorhinus ursinus*) [10]. Unfortunately, in most of the studies summarized in Table 6, the data on the frequency and lesion severity of TMJ-OA is not broken down according to age classes, but only given for the whole sample. Except for the California sea lion (*Zalophus californianus*) it is, however, stated that adult individuals show TMJ-OA lesions significantly more often than juveniles. Differences between sexes were only described for the walrus (*Odobenus rosmarus*), where, like in our study, males exhibited a higher prevalence of TMJ-OA than females [13].

**Table 6:** Frequency and lesion severity of TMJ-OA in different marine carnivorans in relation to age classes

	Individuals examined	TMJ-OA frequency [%]	TMJ-OA severity [%] ^a^			Age class ratio		Affected [%]	
(Sub)species			mild (1)	moderate (2)	severe (3)	(juvenile:adult)		juveniles	adults
*Callorhinus ursinus* [10]	145	20.0	43.9	31.6	24.6	1.5	87:58	n/a ^b^	
*Odobenus rosmarus* [13]	76	60.5	54.7	30.2	15.1	0.3	18:58	11.1	75.9
*Phoca v. richardii* [11]	194	34.5	54.1	28.1	17.8	0.9	93:101	n/a ^b^	
*Phoca v. vitulina* [this study]	1787	53.9	68.1	26.0	5.9	1.0	910:877^c^	24.5 ^c^	84.4
*Zalophus californianus* [12]	497	63.5	82.0	7.0	11.0	0.5	173:324	52.6	69.6
*Enhydra lutris nereis* [14]	1008	4.1	41.5	19.5	39.0	n/a		*n = 6*	*n = 35*
*Ursus maritimus* [15]	249	9.2	52.9	41.2	5.9	1.0	124:125	1.6	11.3

^a^ all affected articular surfaces combined, except for *Z. californianus* (based on individuals affected)

^b^ the study mentioned that adult individuals were statistically significantly more often affected by TMJ-OA than juveniles

^c^ to be comparable with the other studies, the individuals of the juvenile/subadult age class of the present study are here listed as juveniles

Studies in the California sea otter (*Enhydra lutris nereis*) [14], which is the only tool-using marine mammal, and the polar bear (*Ursinus maritimus*) [15] reported lower prevalence of TMJ-OA compared to the analysed pinniped species. The causes for this are presently unclear. Differences in prey spectrum and prey processing compared to pinnipeds, and resulting differences in loading patterns of the TMJ in the two non-pinniped species could be of importance here. However, when looking at prey types and preferred prey-techniques of the studied pinnipeds [50], no obvious relationship between prey habits and prevalence and severity of osteoarthritic lesions is apparent within this group. It may be assumed that differences in the prevalence of TMJ-OA are also influenced by different prey-availabilities, e.g. the proportions of different size classes of prey or of hard-shelled prey in the animals’ diet.

When comparing frequencies of TMJ-OA among (sub-)species, the age composition of the analysed samples is important, as higher frequencies of TMJ-OA tend to be associated with a higher proportion of adult individuals in the study samples (Table 6). As TMJ-OA is a primarily degenerative disease, a higher prevalence of the condition in study samples with a higher proportion of older individuals is expected. If precise information of the age composition of the study samples is lacking, interpretation of the differences in TMJ-OA prevalence among these samples is difficult. Unfortunately, only in the present study the precise age of all studied individuals was known.

In pinnipeds, there is an evolutionary shift of jaw function away from the typical tearing mastication of carnivorans towards a piercing one. Seals catch and fix their prey, and then swallow it down whole. Adoption of this mode of feeding went along with a simplification of the dentition [50]. In the case of large prey, there is a wide opening of the mouth, which may lead to an excessive strain on the TMJ and an anterior displacement of the articular disc. In humans, this has been associated with TMJ-OA [22]. The causes underlying the development of TMJ-OA in pinnipeds remain, however, elusive [13].

Pathological alterations of the TMJ have recently also been studied in terrestrial carnivorans, again based on the assessment of dry skulls from museum collections. In the American black bear (*Ursus americanus*), about half of the studied individuals showed lesions consistent with TMJ-OA [17]. The lesions were all classified as mild, and males were more frequently affected than females [17]. In contrast, only 20% of the examined California mountain lions (*Puma concolor cougar*) showed lesions consistent with TMJ-OA [16]. At present, the causes underlying the variation in frequency and severity of TMJ-OA among different carnivoran species are unclear, and further research into the aetiology and pathogenesis of the condition in pinnipeds and terrestrial carnivorans is needed.

While TMJ-OA is primarily an age-related condition, it can also occur secondary to trauma or other impacts (and their biomechanical consequences) that are unrelated to age. Pathological alterations of the TMJ can for example be induced or accelerated by tooth fractures or loss of one or more teeth that cause modifications of the normal occlusal pattern. Studies on humans and other mammals have demonstrated that even minor disturbances of the normal motion sequence can result in (irreversible) deterioration of the joint [51–53].

Our findings in the European harbour seal tend to confirm the conclusions from these previous studies. While age was the major predictor for the occurrence of TMJ-OA, there were also weak positive correlations between the severity of the condition and the number of fractured teeth and of intravitally lost teeth. In contrast, a significant association between the severity of periodontitis and TMJ-OA lesion severity was not found. We assume that mild and moderate degrees of periodontitis don’t have marked effects on the TMJ, while advanced periodontitis with loss of teeth can cause joint impairment.

The mostly bilateral occurrence of TMJ-OA in the studied harbour seals suggests a predominantly systemic causation of the condition. Only 4.4% of the affected individuals exhibited unilateral TMJ-OA, with a tendency for males to exhibit more pronounced differences between sides than females. This may indicate a role of inter-male aggressive behaviour as a (minor) contributor to the occurrence of TMJ-OA.

Comparison of the findings from our study on the Eastern Atlantic harbour seal with the results previously reported for the Eastern Pacific harbour seal [11] revealed significant differences in prevalence (lower in *P. v. richardii*) and severity of TMJ-OA (higher in affected individuals of *P. v. richardii*) between the two subspecies. As the precise age of the specimens studied by Aalderink et al. [11] was unknown, the reasons for these differences are currently unclear. It is assumed that differences in age composition between the two samples are of great importance in this respect. However, variation in exposure to environmental contaminants may also affect the differences in prevalence. Organohalogen compounds in particular are known to act as endocrine disruptors in mammals, exercising, among others, effects on bone and cartilage metabolism through different pathways [54–56]. High pre- and early postnatal exposure to these substances during skeletal development may pose a risk for permanent damage [57, 58].

During the collection period of our study sample, the North Sea was highly contaminated by both persistent organic pollutants (POPs) and heavy metals and, in consequence, the pollutant burden on the resident harbour seals was high [59–62]. Also in more recent years, high concentrations of legacy POPs have been reported in harbour seals from this area compared to conspecifics from other geographical regions [63–65]. Therefore, it may be speculated that a higher POP exposure of the Eastern Atlantic compared to the Eastern Pacific harbour seals could be a factor contributing to the higher prevalence of TMJ-OA in the former. Previously, an increased prevalence of bony lesions of the skull was observed in grey seals (*Halichoerus grypus*) and harbour seals from the Baltic sea collected during the 1960s (late 1950s in the harbour seals) to 1980s, compared to specimens from the same area collected earlier, i.e., prior to the onset of pollution [66, 67]. The condition of the TMJ was not evaluated in the grey seal studies. Further work is needed to analyse a possible role of POPs in the aetiology and pathogenesis of TMJ-OA in seals.

## Conclusions

The present study, for the first time, analyses the prevalence and severity of TMJ-OA in the Eastern Atlantic harbour seal from the German North Sea. For that, we investigated the spectrum of osteoarthritic lesions of the TMJ in a collection of dry skulls from 1872 individuals using a standardized scoring system. Lesions consistent with TMJ-OA were found in 53.9% of the studied skulls from juvenile/subadult and adult individuals, indicating that TMJ-OA is a common condition in the Eastern Atlantic harbour seal. The dominant predictor of lesion severity was individual age, consistent with the fact that TMJ-OA is a primarily degenerative disease. Lesions severity was furthermore weakly associated with the number of fractured and of intravitally lost teeth. While males were affected more frequently than females, lesion severity tended to be higher in the latter, which may be related to the higher average age at death of the females. Our results demonstrate that the systematic analysis of larger samples of skeletons from museum collections can substantially contribute to the characterization of the health status of the populations of wild mammals.

## Methods

**Fig. 8:**
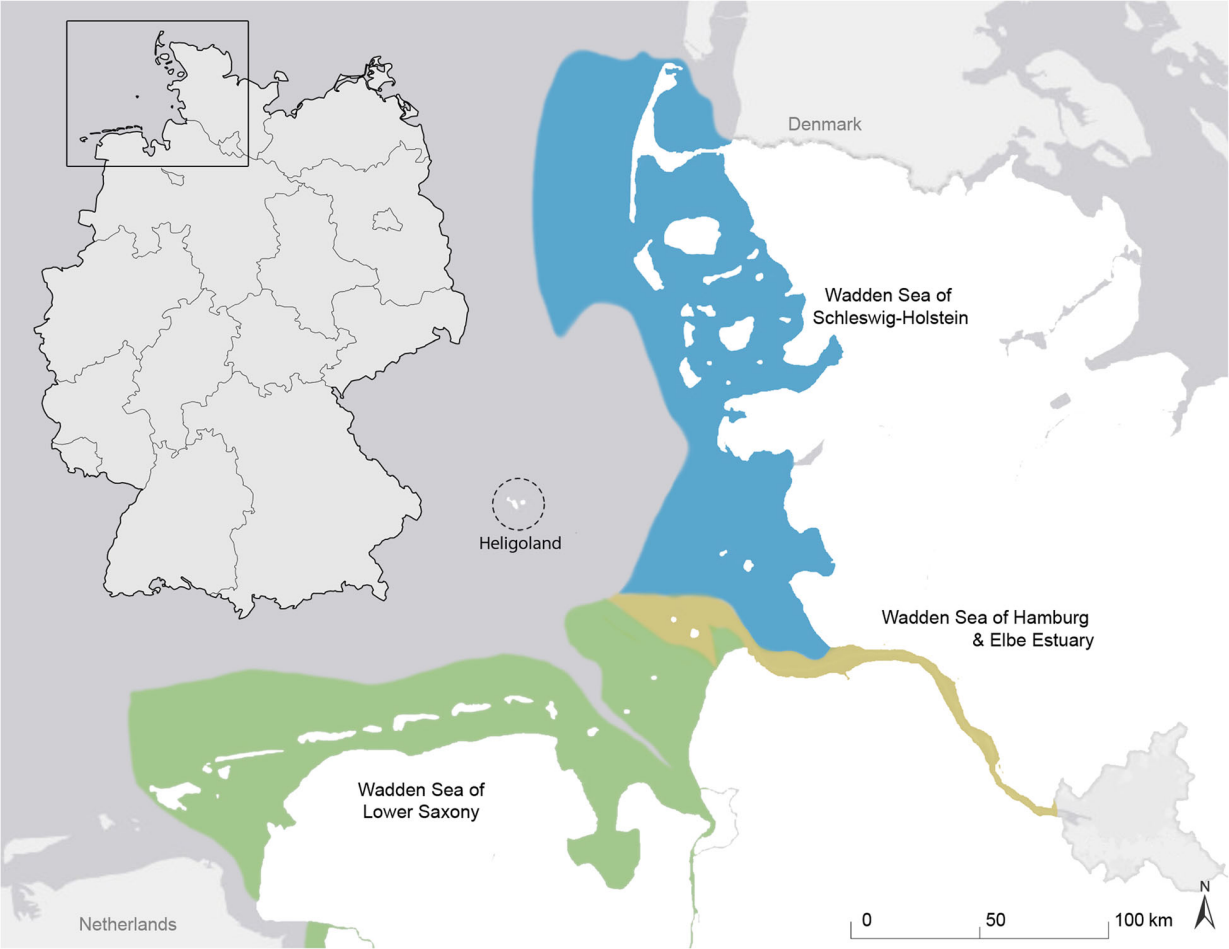
Origin of the *Phoca v. vitulina* individuals, whose skulls were analysed in the present study. Most of the specimens (94.2%) originated from the Wadden Sea of Schleswig-Holstein (*n* = 1680), while the other areas (Wadden Sea of Lower Saxony: *n* = 78, Wadden Sea of Hamburg and Elbe Estuary: *n* = 22, Heligoland: *n* = 7) contributed only small numbers (basemap adapted from Esri, HERE, DeLorme, MapmyIndia, ©OpenStreetMap contributors, and the GIS user community. The overview map of Germany is attributed to David Liuzzo under Creative-Commons-Licence)

We performed a macroscopic examination of 1872 dried skulls of Eastern Atlantic harbour seals from the collection of the Zoological Institute of Kiel University, Germany (ZIK). In all studied skulls, both TMJs (i.e., four articular surfaces) were available for study, and thus in total 7488 articular surfaces (and their margins) were evaluated. Each skull (cranium and mandibles) had previously been labelled with a unique catalogue number. Information on sex, age [41], and the date and locality of collection were given on a tag attached to the skull. Based on average ages at weaning (c. 4–6 weeks) and at reaching sexual maturity (about 5 years) in harbour seals, each specimen was assigned to one of three age classes: (1) neonatal/early postnatal (up to 4 weeks of postnatal age), (2) juvenile/subadult (5 weeks to 5 years of age) and (3) adult (more than 5 years of age). All individuals included in the study had been found dead along the shores of the German North Sea, with the majority (94.2%) originating from the Wadden Sea of Schleswig-Holstein (Fig. 8). Years of collection ranged from 1961 to 1994. Most of the analysed individuals (75.3%) had died during the PDV-epizootic in 1988.

External inspection for gross lesions consistent with TMJ-OA was performed on both mandibular heads and both mandibular fossae of a skull specimen. Using a semi-quantitative scoring scheme, each articular surface (and its margin) was assigned a lesion score of either 0 (normal, no bony lesions of TMJ-OA) or 1 to 3, with higher numbers denoting increasing severity of lesions (Table 7).

**Table 7:** Scoring scheme for evaluating severity of TMJ-OA lesions by macroscopic inspection of articular surfaces (modified after [11])

Score		Criteria
0	normal	smooth articular surface, smooth articular margin, no irregularities
1	mild	slight unevenness of articular surface, presence of small isolated lytic lesions of subchondral bone (pits) affecting less than 25% of the articular surface, articular margin may be locally flattened or slightly thickened, single periarticular osteophytes may be present
2	moderate	more pronounced unevenness of articular surface giving it a bumped appearance, more frequent and/or more profound lytic lesions of subchondral bone affecting 25% to 50% of the articular surface, more pronounced flattening or thickening of articular margins, presence of periarticular osteophytes (at least two features must apply)
3	severe	very pronounced to massive unevenness of articular surface, lytic lesions of subchondral bone affecting more than 50% of the articular surface, markedly irregular articular margin sometimes leading to an abnormal contour of the mandibular fossa and/or the mandibular head that can cause joint incongruity, pronounced periarticular osteophytosis, changes can lead to partial or complete ankylosis of the TMJ (at least two features must apply)

For statistical analysis, the TMJ condition of each skull specimen was classified in two ways. First, the maximum (worst) score (maxTMJ-OA) of the four analysed components (two mandibular heads and two mandibular fossae) was used as the score characterizing an individual. Second, the sum of all four scores obtained on a skull (sumTMJ-OA) was used to characterize an individual. The sum scores were then grouped as follows: 0 (normal), 1–4 (mild TMJ-OA), 5–8 (moderate TMJ-OA) and 9–12 (severe TMJ-OA). The stages normal (no TMJ-OA), mild, moderate, and severe therefore can be used for both max TMJ-OA and sumTMJ-OA. Several skulls of neonatal/early postnatal individuals exhibited an increased porosity of their mandibular heads and/or fossae that represented artificial damage from the maceration process of the skulls and was not considered a pathological condition. This type of post-mortem damage was not observed in the skulls of older individuals.

All statistical analyses were performed using the SPSS software package.^1^ In all tests, *p*-values < 0.05 were considered to indicate statistical significance. The two-tailed t-test for independent samples was used to test for differences in age at death between the two sexes within the age classes. Differences in the prevalence of TMJ-OA between juvenile/subadult and adult individuals and between sexes were assessed using the Chi-square test of independence. The Mann-Whitney-U-test (two-tailed) was used to test for differences in the severity of TMJ-OA between affected (at least one articular surface with a score > 0) juvenile/subadult and adult as well as male and female individuals. Additionally, odds ratios and effect sizes (Cohen’s d for non-parametric tests) were calculated using freeware online-calculators.^2^^,^^3^

The relationship between age at death and severity of TMJ-OA was analysed by calculating Spearman rank correlations. Partial rank correlations, controlling for age at death as a confounder, were calculated to determine possible influences of the severity of periodontal disease, the number of fractured teeth and the number of intravitally lost teeth on the severity of TMJ-OA. Severity of periodontal disease in the analysed skulls was determined using a scoring scheme based on Aalderink et al. [10] that, comparable to the TMJ scoring, distinguishes between a normal condition and three stages of increasingly severe alveolar bone changes. As with TMJ-OA, both the highest lesion score of a jaw quadrant of an individual (maxPeriodontitis) as well as the sum of the scores for the four quadrants (sumPeriodontitis) were used in the analyses. Details of the recording of periodontal lesions, intravitally lost and fractured teeth as well as the results of these investigations will be given elsewhere (Kahle et al., unpublished work).

The occurrence of asymmetric TMJ-OA was determined based on the difference between the highest lesion scores on the left and the right side of an individual. A difference of two or more scores between sides was considered a case of asymmetric TMJ-OA. Differences in the prevalence and severity of TMJ-OA between the Eastern Atlantic harbour seal (data from this study) and the Eastern Pacific harbour seal (data from [11]) were evaluated using the Chi-square test of independence. Aalderink et al. 2015b [11] also used a scoring scheme with 4 scores (normal, mild OA, moderate OA, severe OA). Although their criteria used to assign a lesion score to the TMJ are not identical to those applied in the present study, we nevertheless suggest that a meaningful comparison of the findings from the two studies is possible, and that this also holds for the other studies on North American carnivorans [10, 12–17].

For analysis of the structural changes in the subchondral bone of individuals showing severe TMJ-OA lesions on macroscopic inspection, the mandibular head was cut from the left mandible of an affected individual (ZIK - 29511) and embedded in epoxy resin.^4^ A dorsal view of this mandibular head is shown in Fig. 3 (lowermost specimen in left row). The embedded mandibular head was then sagittally sectioned, and one of the cut surfaces was smoothed and polished as described previously [68]. The uncoated polished surface was viewed in a scanning electron microscope (SEM)^5^; operated in low pressure mode at 20 kV accelerating voltage, using a backscattered electron (BSE) detector.

## Additional file

Additional file 1: Data sheet with information on the studied harbour seals. (XLSX 253 kb)

## Abbreviations

BSE: Backscattered electron
CI: Confidence interval
MaxPeriodontitis: Maximum lesion score of periodontitis in a jaw quadrant of an individual
MaxTMJ-OA: Maximum lesion score of temporomandibular osteoarthritis in the articular surfaces of an individual
OA: Osteoarthritis
PDV: Phocine distemper virus
POPs: Persistent organic pollutants
SEM: Scanning electron microscope
SumPeriodontitis: Combined lesion score of periodontitis based on the summed scores of the four jaw quadrants of an individual
SumTMJ-OA: Combined lesion score of temporomandibular osteoarthritis based on the summed scores of the four articular surfaces of an individual
TMJ: Temporomandibular joint

## Footnotes

1 IBM SPSS Statistics v.24

2
http://vassarstats.net/odds2x2.html

3
https://www.psychometrica.de/effect_size.html#nonparametric

4 Biodur E12, Biodur Products, Heidelberg, Germany

5 Zeiss Evo Ma 15, Carl Zeiss AG, Oberkochen, Germany
